# Gender-specific psychological and social impact of COVID-19 in Pakistan

**DOI:** 10.1192/bjo.2021.1062

**Published:** 2021-12-06

**Authors:** Fauziah Rabbani, Hyder Ali Khan, Suneel Piryani, Areeba Raza Khan, Fahad Abid

**Affiliations:** Department of Community Health Sciences and Office of Research & Graduate Studies, Aga Khan University, Pakistan; Department of Community Health Sciences, Aga Khan University, Pakistan; Department of Community Health Sciences, Aga Khan University, Pakistan; Office of Research & Graduate Studies, Aga Khan University, Pakistan; Department of Psychiatry, Jinnah Postgraduate Medical Centre, Pakistan

**Keywords:** Risk perception, anxiety disorders, depressive disorders, COVID-19, low- and middle-income countries

## Abstract

**Background:**

COVID-19 has rapidly spread across the world. Women may be especially vulnerable to depression and anxiety as a result of the pandemic.

**Aims:**

This study attempted to assess how gender affects risk perceptions, anxiety levels and behavioural responses to the COVID-19 pandemic in Pakistan, to recommend gender-responsive health policies.

**Methods:**

A cross-sectional online survey was conducted. Participants were asked to complete a sociodemographic data form, the Hospital Anxiety and Depression Scale, and questions on their risk perceptions, preventive behaviour and information exposure. Multiple logistic regression analysis was used to assess the effects of factors such as age, gender and household income on anxiety levels.

**Results:**

Of the 1391 respondents, 478 were women and 913 were men. Women considered their chances of survival to be relatively lower than men (59% *v*. 73%). They were also more anxious (62% *v*. 50%) and more likely to adopt precautionary behaviour, such as avoiding going to the hospital (78% *v*. 71%), not going to work (72% *v*. 57%) and using disinfectants (93% *v*. 86%). Men were more likely to trust friends, family and social media as reliable sources of COVID-19 information, whereas women were more likely to trust doctors.

**Conclusions:**

Women experience a disproportionate burden of the psychological and social impact of the pandemic compared with men. Involving doctors in healthcare communication targeting women might prove effective. Social media and radio programmes may be effective in disseminating COVID-19-related information to men.

The COVID-19 virus was first detected in December 2019 in Wuhan, China.^1^ On 11 March 2020, the World Health Organization declared the disease a global pandemic. Since its emergence, there are more than 15.2 million confirmed global cases of the virus, with the number of deaths exceeding 623 000 as of 23 July 2020. In the initial stages of the outbreak, most cases of COVID-19 that were exported internationally had a history of prior travel to Wuhan.^2^ Despite its close geographic proximity with China and Iran, the first two cases of COVID-19 in Pakistan were reported on 26 February 2020.^3^ To curb the spread of the virus, provincial governments in Pakistan initiated partial followed by complete lockdowns in their respective administrative territories. These measures, however, were taken in phases, with educational institutions across the country closing on 13 March 2020, in response to the pandemic.^4^ As of 23 July 2020, Pakistan has over 260 000 confirmed cases of COVID-19, and approximately 5700 deaths.^1^ One year on, Pakistan has experienced four waves of the pandemic, with a total number of 1.2 million cases and 26 000 deaths.^5^

Exposure to a traumatic event, such as a global health crisis, is associated with an increased incidence of anxiety and depression.^6^ Moreover, the stigma and isolation associated with infectious diseases could generate anxiety.^7^ A study conducted on a sample of severe acute respiratory syndrome (SARS) survivors in Hong Kong revealed increased levels of psychological distress and anxiety, not only during the epidemic but also 1 year following the outbreak.^8^ Another study concluded that SARS had long-term psychiatric effects on survivors, with post-traumatic stress disorder (PTSD) and depressive disorders being the most prevalent conditions recorded.^9^ Moreover, a survey conducted in the USA during the H1N1 pandemic, with 7236 participants, suggests an increase in the prevalence of anxiety.^10^

The COVID-19 pandemic is expected to affect women's health and increase their short- and long-term needs for livelihood support and health.^11^ A study conducted in the heavily affected areas around Wuhan highlighted an increase of 7% in the prevalence of post-traumatic stress symptoms (PTSS), and women had significantly higher levels of PTSS than men.^12^ During the SARS epidemic, people with higher levels of anxiety were frequently found to adopt precautionary behaviour.^13^ In keeping with this, a study conducted in the UK found that women practiced precautionary behaviour, such as hand washing and disinfecting surfaces, more often than men.^14^

Female responses to stress and trauma may be contributing factors toward anxiety. Studies also indicate that gendered responses to trauma contribute to the greater onset of depression and PTSD in women.^15^ Evidence attributes this to women believing that worry is uncontrollable, and may cause anxious thoughts.^16^ How young boys and girls are socialised into their gender roles has an impact on these perceptions. A review discussed how mothers are more likely to converse about their emotional condition with their daughters, compared with their sons.^17^ Further, young boys are conditioned to exercise problem-solving skills for managing their emotions, girls are traditionally granted less autonomy. This increases their dependency on others and reduces their capability to effectively cope with anxious thoughts.^17^ Studies from the Eastern Mediterranean region further highlight this, suggesting that this ineffective coping may result in a higher suicide rate among women.^18^ Indeed, in 2020, there were significantly higher female hospital admissions in Malta compared with 2019, with increased presentations of self-harm/suicidal ideation.^19^

The reviewed literature suggests that women are more prone to anxiety and depression, and that the mental health status of the population tends to deteriorate during large epidemics or pandemics. The current crisis requires policies that facilitate large-scale behaviour change, science communication and strategies to cope with stress through psychological platforms.^20^ Immediate risk assessment and quick action of Vietnamese policy makers, as well as the seamless coordination between government and citizens in implementing protective measures, has resulted in low case numbers and zero deaths in the country. This has been in conjunction with media appropriately promoting public awareness about how people can protect themselves and their communities.^21^

This study, therefore, aimed to identify gender differences in perceived risk, anxiety levels and behavioural responses to COVID-19. This will help to develop gender-responsive policies to mitigate progression toward serious mental health conditions.

## Method

### Study design

This is a cross-sectional study, with the survey tool disseminated online. The first case was reported on 26 February 2020, in Pakistan. The study was conducted between 1 May 2020 and 15 May 2020, during a government-imposed lockdown. Ethical approval was obtained from the Ethical Review Committee of the Aga Khan University, Pakistan (ERC#2020-4806-10421).

### Study participants

A convenience sampling strategy was used to recruit participants. The questionnaire was launched for 2 weeks on the social media pages of a Karachi-based university hospital. Potential study participants were encouraged to share the link on their social media platforms. People aged ≥18 years, residing in Pakistan for the past month, with access to the internet and willingness to participate in the study were included. Participants who could not respond to the study tool in either English or Urdu, and those who reported having filled the questionnaire at least once before, were excluded. This was an online survey tool (Google form), so no verbal or written consent could be taken in the traditional sense. Participants who met the above-stated eligibility criteria and consented to participate were able to further navigate the system. Respondents found ineligible or those not willing to consent were redirected to a thank you message, and further access to the tool was halted by the Google form.

A total of 1406 respondents completed the online questionnaire. Fifteen respondents preferred not to disclose their gender. Thus, 1391 participants were included in this study.

### Data collection

Data was collected through an online self-administered structured questionnaire developed on Google Forms. Respondents were inquired about their gender, age, level of education, household income and city of permanent residence. They were asked how likely it is that they or their families might be infected with COVID-19 if no preventive measures were taken. Further questions assessed how participants perceived the severity of the symptoms caused by COVID-19, their likelihood of survival if infected and their adoption of precautionary measures. Respondents also rated the reliability of various sources of COVID-19-related information. Subsequently, the psychological impact of COVID-19 on respondents’ jobs, personal life, sleep pattern and eating habits was assessed. Participants’ anxiety and depression levels were assessed by the validated Hospital Anxiety and Depression Scale (HADS). This comprises 14 items (seven items on anxiety and seven items on depression) scored on a four-point Likert scale. The lowest possible scores for anxiety and depression are 0, and the highest possible score is 15 for anxiety and 21 for depression. The scale defines a normal score as ≥7, borderline abnormal score as 8–10 and abnormal score as ≤11. Higher scores imply greater severity of anxiety or depression.^22^

### Data analysis

Data collected from respondents was stored in Google Spreadsheets, then imported to Microsoft Excel 2016 and SPSS version 21 for Windows (IBM Corporation). Data was cleaned, coded and analysed with SPSS version 21. A descriptive analysis was performed. Results were tabulated as number (percentage) for qualitative variables and mean (±s.d.) for quantitative variables. Independent *t*-test, Mann–Whitney *U*-test or Pearson *χ*^2^-test was applied to assess the differences between women's and men's perception of susceptibility and severity toward COVID-19, anxiety, depression, the psychological impact of COVID-19, adoption of precautionary measures and reliability of information sources. Responses were classified as ʻmissing' if respondents left the question blank, and was excluded from the analysis. ‘Don't know’ was an option in survey questions that required a response on a five-point Likert scale, wherein 4 and 5 were considered as agree and strongly agree, respectively. Therefore, if the respondents replied ʻDon't know', such responses were considered as a non-agreement on the scale. This type of categorisation for Likert scale-based responses is consistent with the literature.^23^ Bivariate and multiple logistic regression analyses were performed to identify predictors (age, gender and household income) of anxiety and depression. Initially, a single predictor at a time was entered; crude odds ratios and 95% confidence intervals were computed by bivariate analysis. Multivariate analysis with all predictors entered at the same time was completed to adjust for the effect of confounding, and adjusted odds ratios and 95% confidence intervals were computed. All statistical tests were two-sided, and a *P*-value of ≤0.05 was considered statistically significant and indicated in results where appropriate. Exact *P*-values are given where they are >0.05.

## Results

A total of 1391 participant responses were included in the analysis. Table 1 shows the sociodemographic characteristics (age, education and household income) of women and men, which are comparable. The majority of the respondents were aged between 25 and 34 years (69% of women *v*. 66% of men) and possessed a Bachelor's degree or above (75% of women *v*. 79% of men). About 29% of women and 17% of men preferred not to disclose their household income. Around a third of women (32%) and two-fifths of men (40%) mentioned that their household income was below PKR 60 000. More women (69%) than men (40%) from Karachi participated in the survey (Table 1).

**Table 1:** Sociodemographic characteristics of the respondents by gender

Characteristics	Female, *n* = 478, *n* (%)^a^	Male, *n* = 913, *n* (%)^a^	*P*-value^b^
Age, years			
18–24	167 (34.9)	291 (31.9)	0.833
25–34	163 (34.1)	315 (34.5)	
35–44	89 (18.6)	185 (20.3)	
45–54	32 (6.7)	72 (7.9)	
55–64	19 (4.0)	32 (3.5)	
65–74	7 (1.5)	16 (1.8)	
≥75	0	1 (0.1)	
Prefer not to disclose	1 (0.1)	1 (0.1)	
Education			
Intermediate or below	119 (24.9)	188 (20.6)	0.069
Bachelor's or above	358 (74.9)	721 (79.0)	
Prefer not to disclose	1 (0.2)	4 (0.4)	
Household income			
≤PKR 20 000	33 (8.4)	98 (12.0)	0.877
PKR 20 001–40 000	53 (13.5)	133 (16.2)	
PKR 40 001–60 000	40 (10.2)	99 (12.1)	
PKR 60 001–80 000	28 (7.1)	74 (9.0)	
PKR 80 001–100 000	28 (7.1)	58 (7.1)	
PKR 100 001–120 000	33 (8.4)	85 (10.4)	
>PKR 120 000	63 (16.0)	135 (16.5)	
Prefer not to disclose	115 (29.3)	137 (16.7)	
Permanent residence			
Karachi	329 (68.8)	364 (39.9)	<0.001
Lahore	36 (7.5)	91 (10.0)	
Islamabad	25 (5.2)	56 (6.1)	
Peshawar	8 (1.7)	45 (4.9)	
Quetta	2 (0.4)	13 (1.4)	
Hyderabad	18 (3.8)	34 (3.7)	
Other	60 (12.6)	310 (34.0)	

a. Percentages may not total 100 because of rounding.

b. Pearson *χ*²-test.

Around three-fourths of respondents perceived that they (strongly agree/agree: 71% of women *v*. 73% of men; *P* = 0.489) and their family (strongly agree/agree: 74% of women *v*. 73% of men; *P* = 0.599) might be infected with COVID-19 if no preventive measures were taken. However, significantly more women than men considered symptoms of COVID-19 (if infected) as severe (very severe/severe: 46% of women *v*. 39% of men; *P* < 0.05). Further, 59% of women perceived themselves as likely to survive an infection, compared with 73% of men (*P* < 0.001) (Table 2).

**Table 2:** Perceived severity and susceptibility for COVID-19 in women and men

Variable	Female, *n* = 478, *n* (%)^a^	Male, *n* = 913, *n* (%)^a^	*P*-value
Susceptibility			
I might contract the disease if no preventive measure is taken			
Strongly agree	175 (36.6)	336 (36.8)	0.489^b^
Agree	166 (34.7)	329 (36.0)	
Neutral	58 (12.1)	110 (12.0)	
Disagree	25 (5.2)	44 (4.8)	
Strongly disagree	54 (11.3)	85 (9.3)	
Don't know	0	9 (1.0)	
My family might contract the disease if no preventive measure is taken			
Strongly agree	181 (37.9)	352 (38.6)	0.599^b^
Agree	172 (36.0)	314 (34.4)	
Neutral	40 (8.4)	106 (11.6)	
Disagree	24 (5.0)	46 (5.0)	
Strongly disagree	60 (12.6)	85 (9.3)	
Don't know	1 (0.2)	10 (1.1)	
I might contract COVID-19 if one of my family members tests positive for the disease			
Strongly agree	141 (29.6)	273 (29.9)	0.844^b^
Agree	187 (39.2)	333 (36.5)	
Neutral	65 (13.6)	132 (14.5)	
Disagree	25 (5.2)	60 (6.6)	
Strongly disagree	47 (9.9)	84 (9.2)	
Don't know	12 (2.5)	30 (3.3)	
Severity			
Seriousness of symptoms caused by SARS-COVID19			
Very severe	68 (14.2)	79 (8.7)	0.045^b^
Severe	150 (31.4)	275 (30.1)	
Neutral	126 (26.4)	213 (23.3)	
Not severe	39 (8.2)	91 (10.0)	
Not severe at all	25 (5.2)	48 (5.3)	
Don't know	70 (14.6)	207 (22.7)	
Chance of survival if infected with COVID-19			
Very high	67 (14.0)	238 (26.1)	<0.001^b^
High	214 (44.8)	427 (46.8)	
Neutral	137 (28.7)	136 (14.9)	
Not high	27 (5.6)	38 (4.2)	
Not high at all	5 (1.0)	15 (1.6)	
Don't know	28 (5.9)	59 (6.5)	

a. Percentages may not total 100 because of rounding.

b. Mann–Whitney *U*-test.

Women were also reported to have a higher HADS anxiety score (mean ± s.d.: 6.80 ± 3.61 in women *v*. 5.93 ± 3.58 in men; *P* < 0.001). Furthermore, the HADS depression score was high among women (mean ± s.d.: 8.39 ± 3.93 in women *v*. 8.01 ± 3.69 in men; *P* = 0.079). More women were found to be depressed compared with men, with 58% of women and 54% of men (*P* = 0.244) scoring above the depression cut-off point (≥8). Around three-fifths of the respondents (strongly agree/agree: 58% of women *v*. 61% of men; *P* = 0.242) mentioned that COVID-19 had affected their jobs. About three-fourths of the respondents (strongly agree/agree: 73% of women *v*. 74% of men; *P* = 0.263) also expressed concerns that the current pandemic is affecting their personal life. About two-fourths of the respondents believed that their sleeping pattern (strongly agree/agree: 40% of women *v*. 39% of men; *P* = 0.710) and eating habits (strongly agree/agree: 36% for both women and men; *P* = 0.726) have been disturbed because of COVID-19. Significantly more men compared with women mentioned that they might start/increase cigarette consumption (strongly agree/agree: 6% of women *v*. 11% of men; *P* < 0.001), and might resort to the use of recreational drugs such as marijuana, crystallised methamphetamines, cocaine or opium products, etc. (strongly agree/agree: 4% of women *v*. 6% of men; *P* < 0.001) (Table 3).

**Table 3:** Psychological impact ofCOVID-19 among men and women

Variable	Female, *n* (%)^a^	Male, *n* (%)^a^	*P*-value
Anxiety (HADS-A score)			
Normal (<6)	184 (38.5)	455 (49.8)	<0.001^b^
Abnormal (≥6)	294 (61.5)	458 (50.2)	
Mean (±s.d.)	6.80 (3.61)	5.93 (3.58)	<0.001
Depression (HADS-D score)			
Normal (<8)	202 (42.3)	417 (45.7)	0.244^b^
Abnormal (≥8)	276 (57.7)	496 (54.3)	
Mean (±s.d.)	8.39 (3.93)	8.01 (3.69)	0.079
COVID-19 will affect my job			
Agree	278 (58.3)	559 (61.2)	0.242^c^
Neutral	78 (16.4)	149 (16.3)	
Disagree	102 (21.4)	177 (19.4)	
Don't know	19 (4.0)	28 (3.1)	
COVID-19 will affect my personal life			
Agree	301 (63.0)	581 (63.6)	0.263^c^
Neutral	73 (15.3)	171 (18.7)	
Disagree	97 (20.3)	150 (16.4)	
Don't know	7 (1.5)	11 (1.2)	
COVID-19 has affected my sleeping pattern			
Agree	193 (40.4)	352 (38.6)	0.710^c^
Neutral	77 (16.1)	161 (17.6)	
Disagree	190 (39.7)	372 (40.7)	
Don't know	18 (3.8)	28 (3.1)	
COVID-19 has affected my eating habits			
Agree	170 (35.6)	329 (36.1)	0.726^c^
Neutral	90 (18.8)	158 (17.3)	
Disagree	208 (43.5)	399 (43.8)	
Don't know	10 (2.1)	26 (2.9)	
I might start smoking cigarettes/my cigarette consumption might increase			
Agree	28 (5.9)	102 (11.2)	<0.001^c^
Neutral	23 (4.8)	95 (10.4)	
Disagree	411 (86.0)	669 (73.3)	
Don't know	16 (3.3)	47 (5.1)	
I might start/increase the use of recreational drugs (such as marijuana, crystal meth, cocaine or opium products, etc.)			
Agree	21 (4.4)	51 (5.6)	0.001^c^
Neutral	21 (4.4)	69 (7.6)	
Disagree	417 (87.2)	747 (81.8)	
Don't know	19 (4.0)	46 (5.0)	

HADS-A, Hospital Anxiety and Depression Scale – Anxiety; HADS-D, Hospital Anxiety and Depression Scale – Depression.

a. Percentages may not total 100 because of rounding.

b. Pearson *χ*²-test.

c. Mann–Whitney *U*-test.

Significant differences were identified between women and men in adopting several precautionary measures, such as washing their hands with soap/sanitiser frequently (100% of women *v*. 98% of men; *P* < 0.05), wearing masks (93% of women *v*. 92% of men; *P* < 0.05), covering nose and mouth when sneezing or coughing (98% of women *v*. 95% of men; *P* < 0.05), avoiding contacting people who have a fever or respiratory symptoms (95% of women *v*. 91% of men; *P* < 0.05), avoiding going out (87% of women *v*. 71% of men; *P* < 0.001), avoiding crowded areas (96% of women *v*. 92% of men; *P* < 0.01), refraining from going to a hospital or clinic (78% of women *v*. 71% of men; *P* < 0.001), avoiding going to work (72% of women *v*. 57% of men; *P* < 0.001), avoiding social events (97% of women *v*. 93% of men; *P* < 0.05) and avoiding domestic travel (93% of women *v*. 86% of men; *P* < 0.001) (Table 4.).

**Table 4:** Adoption of precautionary measures

Variable	Female, *n* (%)	Male, *n* (%)	*P*-value
Adoption of precautionary measures (frequency of yes response)^a^			
Wear face masks	444 (92.9)	834 (91.3)	0.025^b^
Wash hands frequently (with soap or hand sanitiser)	476 (99.6)	891 (97.6)	0.012^b^
Disinfecting floors and tables at home (with phenyl products)	391 (81.8)	646 (70.8)	<0.001^b^
Cover nose and mouth when sneezing or coughing	468 (97.9)	870 (95.3)	0.043^b^
Avoid contacting people who have fever or respiratory symptoms	452 (94.6)	832 (91.1)	0.040^b^
Avoid contacting people who have been traveling abroad within 1 month	441 (92.3)	800 (87.6)	0.058^b^
Avoid going out	417 (87.2)	646 (70.8)	<0.001^b^
Avoid crowded areas	457 (95.6)	840 (92.0)	0.003^b^
Avoid going to meat shops/market	391 (81.8)	580 (63.5)	<0.001^b^
Avoid going to hospital or clinic	373 (78.0)	648 (71.0)	<0.001^b^
Avoid going to work	346 (72.4)	516 (56.5)	<0.001^b^

a. Multiple answers.

b. Pearson *χ*²-test.

Information about COVID-19 provided by the doctor was considered reliable by significantly more women than men (very reliable/reliable: 91% of women *v*. 88% of men; *P* < 0.05). Most of the respondents (very reliable/reliable: 81% of women *v*. 82% of men; *P* = 0.507) thought that the information provided through official websites, such as those run by the government, was reliable. Significantly more men than women believed that the radio (very reliable/reliable: 46% of women *v*. 55% of men; *P* < 0.05) and family or friends (very reliable/reliable: 46% of women *v*. 55% of men; *P* < 0.05) were reliable sources for gaining information about COVID-19. Furthermore, television (very reliable/reliable: 57% of women *v*. 61% of men; *P* < 0.05); newspapers (very reliable/reliable: 56% of women *v*. 58% of men; *P* = 0.843); magazines (very reliable/reliable: 39% of women *v*. 44% of men; *P* = 0.104); social media, such as Facebook, WhatsApp and Instagram (very reliable/reliable: 28% of women *v*. 32% of men; *P* = 0.459); and unofficial websites (very reliable/reliable: 22% of women *v*. 31% of men; *P* < 0.05) were considered as reliable information sources by more men than women (Table 5).

**Table 5:** Perceived reliability of information sources in Pakistan, by gender

Reliability of information sources	Female, *n* = 478, *n* (%)^a^	Male, *n* = 913, *n* (%)^a^	*P*-value^b^

Newspaper			
Reliable/very reliable	248 (56.0)	512 (58.2)	0.843
Neutral	150 (33.9)	256 (29.2)	
Unreliable/very unreliable	45 (10.1)	111 (12.6)	
Magazine			
Reliable/very reliable	173 (38.5)	389 (44.0)	0.104
Neutral	186 (41.4)	329 (37.2)	
Unreliable/very unreliable	90 (20.0)	167 (18.9)	
Radio			
Reliable/very reliable	207 (46.0)	486 (54.9)	0.014
Neutral	185 (41.1)	284 (32.1)	
Unreliable/very unreliable	58 (12.9)	115 (13.0)	
Television			
Reliable/very reliable	258 (57.3)	541 (61.1)	0.401
Neutral	117 (26.0)	206 (23.3)	
Unreliable/very unreliable	75 (16.7)	138 (15.6)	
Official websites such as that of the Government			
Reliable/very reliable	367 (81.4)	727 (82.2)	0.507
Neutral	61 (13.5)	93 (10.5)	
Unreliable/very unreliable	23 (5.1)	64 (7.2)	
Unofficial websites			
Reliable/very reliable	99 (22.0)	271 (30.7)	0.013
Neutral	138 (30.7)	256 (29.0)	
Unreliable/very unreliable	212 (47.3)	357 (40.3)	
Social media platforms (WhatsApp, Facebook, Instagram)			
Reliable/very reliable	131 (29.2)	286 (32.3)	0.459
Neutral	130 (29.0)	236 (26.6)	
Unreliable/very unreliable	188 (41.8)	364 (41.1)	
Your doctor			
Reliable/very reliable	410 (91.1)	775 (87.6)	0.041
Neutral	33 (7.3)	96 (10.8)	
Unreliable/very unreliable	7 (1.6)	14 (1.6)	
Your family or friends			
Reliable/very reliable	206 (46.0)	471 (53.3)	0.003
Neutral	155 (34.6)	288 (32.6)	
Unreliable/very unreliable	87 (19.4)	125 (14.1)	

a. Percentages may not total 100 because of rounding.

b. Mann–Whitney *U*-test.

Table 6 demonstrates the predictors of anxiety. Gender, age and household income had a significant positive association with anxiety. Women were nearly two times more likely to be anxious than men (adjusted odds ratio 1.70, 95% CI 1.26–2.28, *P* <0.001). Moreover, respondents of a younger age (25–34 years) (adjusted odds ratio 2.30, 95% CI 1.26–4.18, *P* < 0.001) were nearly two times more likely to have anxiety than respondents over 55 years of age. Respondents with a household income between PKR 60 000 and 120 000 were more likely to have anxiety than respondents with a household income of > PKR 120 000 (adjusted odds ratio 1.84, 95% CI 1.27–2.67, *P* < 0.001).

**Table 6:** Predictors of anxiety in Pakistan

Characteristics	Crude odds ratio (95% CI)	*P*-value	Adjusted odds ratio^a^ (95% CI)	*P*-value
Gender				
Female	1.59 (1.27−1.99)	<0.001	1.69 (1.26−2.27)	<0.001
Male	Reference		Reference	
Age, years				
18–24	2.36 (1.65−3.38)	<0.001	2.68 (1.70−4.23)	<0.001
25–34	2.57 (1.80−3.68)	<0.001	2.77 (1.82−4.22)	<0.001
35–44	2.42 (1.64−3.56)	<0.001	2.53 (1.61−3.99)	<0.001
≥45	Reference		Reference	
Household income				
≤PKR 60 000	1.69 (1.20−2.36)	0.002	1.49 (1.05−2.11)	0.027
PKR 60 001–120 000	1.85 (1.29−2.65)	0.001	1.84 (1.27−2.67)	0.001
>PKR 120 000	Reference		Reference	

a. Adjusted for gender, age, education and household income.

Table 7 shows the predictors of depression. Only household income was found to have a significantly positive association with depression in multivariate analysis. Respondents having a household income of PKR 60 000–120 000 were more likely to have anxiety compared with respondents who had a household income of >PKR 120 000 (adjusted odds ratio 1.99, 95% CI 1.38–2.87).

**Table 7:** Predictors of depression in Pakistan

Characteristics	Crude odds ratio (95% CI)	*P*-value	Adjusted odds ratio^a^ (95% CI)	*P*-value
Gender				
Female	1.15 (0.92−1.44)	0.224	1.28 (0.96−1.71)	0.087
Male	Reference		Reference	
Age, years				
18–24	1.48 (1.04−2.09)	0.027	1.26 (0.81−1.95)	0.305
25–34	1.34 (0.95−1.89)	0.093	1.32 (0.88−1.97)	0.183
35–44	1.42 (0.97−2.07)	0.069	1.44 (0.93−2.24)	0.103
≥45	Reference		Reference	
Household income				
≤PKR 60 000	1.57 (1.12−2.19)	0.009	1.53 (1.09−2.17)	0.015
PKR 60 001–120 000	1.99 (1.39−2.85)	<0.001	1.99 (1.38−2.87)	<0.001
>PKR 120 000	Reference		Reference	

a. Adjusted for gender, age, education and household income.

## Discussion

This study assessed how gender roles in Pakistan can affect anxiety levels and behavioural responses among men and women during the COVID-19 pandemic. Both men and women were found to be anxious because of the COVID-19 pandemic. However, compared with men, more women perceived the disease to be fatal, and women were more likely to engage in preventive behaviour. These results highlight a greater need to develop gender-responsive policies in the fight to contain COVID-19.

Overall, fewer women than men responded to the questionnaire. This may be because of the male-dominated access to internet facilities in Pakistan. In Pakistan, cellular devices remain the most frequent means of accessing internet facilities, and there is a gender gap of 38% in mobile phone ownership. Indeed, several reports on internet penetration found that at least three-fourths of internet and social media users in the country are male.^24^

There were also significant differences in respondents’ cities of permanent residence. Three-fifths of all of the female participants and two-fifths of the male participants were from Karachi. This was expected, as the survey tool was disseminated over a Karachi-based university hospital's Facebook page. There are significant provincial disparities in access to internet facilities. The fewest respondents were from Balochistan, further reflecting the province's poor internet accessibility. More male (a third) respondents were from smaller cities and towns throughout the country, compared with a tenth of female respondents. These differences can be attributable to fewer women having mobile phone ownership and social media usage in smaller towns/cities.^24^

Although men and women considered themselves equally susceptible to a COVID-19 infection, women were more likely to perceive the disease to be fatal and worry about their own and their family's healthcare. Apart from their professional role, women serve as primary caregivers within their family.^25^ Women's greater sensitivity toward familial roles and responsibilities was also reflected in a European study, which noted that pregnant women had heightened stress levels regarding the health status of their older relatives, children and unborn babies during COVID-19.^26^ Similarly, a comparison of COVID-19-related content shared on Twitter by men and women based in the USA found that women were more likely to tweet about family, social distancing and healthcare, whereas men were more likely to tweet about sports cancellations and politics.^27^

Men's casual attitude can be explained by gender-disaggregated data (until 24 June 2020) on COVID-19 in Pakistan, which shows that three-quarters of diagnosed cases and deaths were among men compared with a quarter among women.^28^ Gender-specific patterns of smoking are implicated as a significant contributor to disease severity among men.^29^ It was also noted that men in our study had a higher likelihood to start smoking cigarettes and using recreational drugs during the pandemic, compared with women. It is therefore not surprising that excess mortality during the pandemic was higher among men complemented by comorbid non-communicable diseases and a delay in seeking lifesaving care.^30,31^

Unequal share of household responsibilities and women staying home because of school closure, lockdown and work-from-home orders may have resulted in women being more stressed. Housework is largely undocumented and unpaid.^32^ Working mothers spend more hours engaged in household work and child care than their husbands. One study conducted in the UK during the lockdown estimates that, on average, mothers spend 11 hours more per week on child care than fathers. Single mothers have less time to spend on child care than partnered mothers, as they are single-handedly forced to bear the brunt of the shifts in the job market.^33^ This additional housework could result in women permanently exiting from the labour market and add to their anxieties. These developments are concerning and emphasise the urgent need to develop labour policies that protect women in the workforce.

Despite being at lower risk of exposure to the disease and subsequently succumbing to it, women in this study were more likely to practice hygiene measures and social distancing, such as avoiding going to meat shops/markets, going out and going to work. The Gallup survey conducted during various waves of the pandemic (April 2020 to August 2021) shows that Pakistani women are more likely to stay at home as a preventive measure.^34^ One digital ethnographic study in Pakistan suggests that one in four men reported having attended Friday (congregational) prayers during the early phase of the pandemic in 2020.^35^ Although this finding might imply that men are considerably less interested in social distancing practices, men constitute a majority of waged and salaried workers in cities, and only a fifth of Pakistani women are part of the labour force.^36^ These differences in employment could explain why men are less likely to conform to social distancing practices than women.

Women were more likely to report that they avoid going to hospitals during the pandemic as a measure of social distancing. It may be too soon to estimate the impact of COVID-19 on maternal and child health services, but one study estimates that a modest decline of 10% in coverage of pregnancy-related and new-born healthcare in low-and middle-income countries could result in an additional 1.7 million pregnant women and 2.6 million new-borns in need of urgent medical care.^37^ Research conducted during the 2013–2016 Ebola outbreak in Western Africa shows how sexual and reproductive health was adversely affected by strains on healthcare systems, which often resulted in interruptions of care and redirected resources.^38^ A similar reduction in access can be seen during the current pandemic. Clinics operated by Marie Stopes International, which is the largest private provider of family planning services in Pakistan, reports that its activities have been reduced by up to 40% in Pakistan, as a result of the pandemic.^39^ Furthermore, some studies noted how the diversion of staff and funding from maternal, neonatal and child health programmes to the front line of the COVID-19 response has also decreased the quality of services available to women.^40,41^

Although women refrained from accessing health facilities during the pandemic, as a measure of social distancing, they still considered doctors as the most reliable source of information on COVID-19. This is consistent with findings from the Gallup survey in Pakistan.^34^ Hence, social media awareness campaigns and telemedicine engaging doctors can target women who tend to be more socially isolated than men during the pandemic. A study in Karachi has also commented on the need to develop psychosocial support, including essential assistance through online support groups, awareness through television or social media, and telemedicine for women.^42^

In this study, women showed higher levels of anxiety and depression compared with men, which suggests that they hold a greater psychiatric burden of the COVID-19 pandemic. One study conducted in China established that 54% of respondents suffered some psychological effect from the outbreak.^43^ This Chinese study found that women have suffered a greater psychological effect as a result of the pandemic compared with men, and may be three times more anxious than their male counterparts. Similarly, our findings corroborate with data from Turkey, where women had significantly higher scores of depression and anxiety.^44^ Surely this indicates that women have a higher vulnerability for developing anxiety disorders.

This is a novel study accounting for differences in perceptions of men and women, with regards to risk perceptions, anxiety levels and behavioural responses to COVID -19. However, the study had some limitations. The survey could only be accessed by the literate population in Pakistan with access to internet, so the generalisability of results needs to be viewed with caution. Moreover, in this study, most of the respondents were aged <35 years, which may not accurately represent the views of the older population who are at greater risk of contracting COVID-19. Nevertheless, as the majority of the population in Pakistan is <30 years of age, the responses are likely to represent the perceptions of the literate general population in Pakistan. Additionally, the perceptions are related to the early phase of the pandemic, and the trajectory of COVID-19-related beliefs over time is yet to be determined. However, the anxieties around COVID-19 still prevail. Indeed, the latest Gallup survey (2021) in Pakistan shows that approximately half of Pakistanis in urban areas are worried if people around them do not wear masks in public.^34^ Moreover, a large survey assessing anxiety and depression symptoms in the USA found that women were equally anxious at the start of the pandemic as they were in August 2021.^45^ Hence, the recommendations of our study to develop gender-responsive psychosocial support strategies for women, and provide additional support in their employment and child care, are needed more than ever, as we continue to battle with the pandemic.

In summary, this study assessed the gender differences in risk perceptions (susceptibility and severity of the disease), preventive behaviour (social distancing, enhanced hygiene measures) and anxiety levels during the early phase of COVID-19. The results highlight the need for gender-responsive policies in mitigating the health and economic impact of the COVID-19 pandemic. The global economy has ground to a stop, and respondents face severe economic uncertainty. Differences in type of work based on gender may result in men being unable to maintain social distancing. Furthermore, it results in women being burdened with increasing housework, which can affect their ability to engage in professional work and add to their stresses. This indicates the urgent need to develop labour laws to protect the workforce, particularly women. Furthermore, the results indicate potential avenues of disseminating gender-specific health communication. Involving doctors in healthcare communication targeting women, focusing on their need to avoid skipping hospital appointments, might prove effective. Research is required to assess strategies of reducing the frequency of in-person maternal, neonatal and child health appointments, and the potential of telemedicine for all women to remain in contact with the health system. As men are more likely to trust what they read on social media, especially if it is shared by friends or family, social media campaigns and radio programming may be effective in disseminating information, and the latter could be an effective tool to diverse audiences.^46^ This health communication should include messages about men's higher risk of dying owing to COVID-19, a lack of non-communicable disease management and smoking cessation. Most importantly, based on the discussion, policy measures must be taken to ensure the continued provision of quality healthcare to women.

Differences in type of work based on gender may result in men being unable to maintain social distancing. Furthermore, it results in women being burdened with increasing housework, which can affect their ability to engage in professional work.

## Data Availability

Data and materials from the study are in the custody of Aga Khan University. Anonymised data that support the findings of this study are available from the corresponding author, F.R., upon reasonable request.

## Supplementary material

For supplementary material accompanying this paper visit http://dx.doi.org/10.1192/bjo.2021.1062. click here to view supplementary material
